# A deep learning–based study of player styles and cross-league performance adaptation mechanisms: a case study of the NBA and CBA

**DOI:** 10.3389/fspor.2025.1639972

**Published:** 2025-12-04

**Authors:** Yunhan Xiao, Weipin Li, Jiangang Chen

**Affiliations:** School of Sport and Health Science, Xi'an Physical Education University, Xi'an, Shaanxi Province, China

**Keywords:** deep learning, player style1, cross-league athletic performance, adaptation mechanism, NBA;CBA, interpretable analysis

## Abstract

**Introduction:**

This study explores how deep learning and interpretable modeling can reveal the impact of player styles on performance across basketball leagues. By examining stylistic features and their influence on cross-league adaptation, the research aims to provide a quantitative framework to understand performance mechanisms in diverse competitive environments.

**Methods:**

Game data from players and teams spanning the 2019–2024 seasons were collected as samples. The study first clustered and modeled players' technical styles using principal component analysis (PCA), t-distributed stochastic neighbor embedding (t-SNE), and Gaussian mixture models. It then employed a branch-type multilayer perceptron (Branch-MLP), combined with the SHAP (SHapley Additive exPlanations) algorithm, to conduct interpretable analyses of mainstream tactical structures.

**Results:**

Findings reveal that the NBA prioritizes offensive efficiency and coordinated team play, whereas the CBA emphasizes ball-possession control and physical confrontation. The Branch-MLP model demonstrated high accuracy in tactical recognition tasks. Quantitative evaluations further showed that interior defense-focused role players maintained more stable performance across leagues, while perimeter ball-handling players exhibited greater variability.

**Discussion/Conclusion:**

This study advances the quantitative analysis of athletic performance by integrating deep learning with interpretable analytics. Its insights provide actionable references for training, player transfers, and youth talent development, supporting data-driven decisions in professional basketball management.

## 1 Introduction

In recent years, the globalization of professional sports has accelerated, and the cross-league movement of professional players has become increasingly common—an important trend in international basketball. More and more athletes choose to develop across leagues and borders in search of higher-level competition and career opportunities (1). Top-tier leagues are growing more reliant on foreign players, who stand out in key metrics such as playing time and scoring efficiency, prompting clubs to favor recruiting overseas talent and thereby hastening the global flow and integration of basketball talent (2). However, pronounced differences in playing styles among leagues create considerable uncertainty in players' performance after transfers, posing a prominent challenge to current international basketball exchange and integration.

Against this backdrop, the National Basketball Association (NBA) and the Chinese Basketball Association (CBA)—two of the world's most influential and representative leagues—have seen even more frequent cross-league player movement, along with more pronounced adaptation issues. On the one hand, some young CBA elite players actively attempt to enter the NBA through its Summer League, training camps, and other channels, yet very few manage to secure a stable foothold and long-term success. On the other hand, many NBA players—due to injuries, career bottlenecks, or being in the later stages of their careers—opt to join the CBA or other overseas leagues. Existing studies indicate that although these players possess high-level skills, they often face multiple challenges in the new league, including cultural differences, divergent competitive systems, role adjustments, and psychological adaptation, leading to performance fluctuations (3–5). Thus, how players can maintain and enhance their performance in a new league environment has become a problem that professional basketball must address.

**Figure 1:**
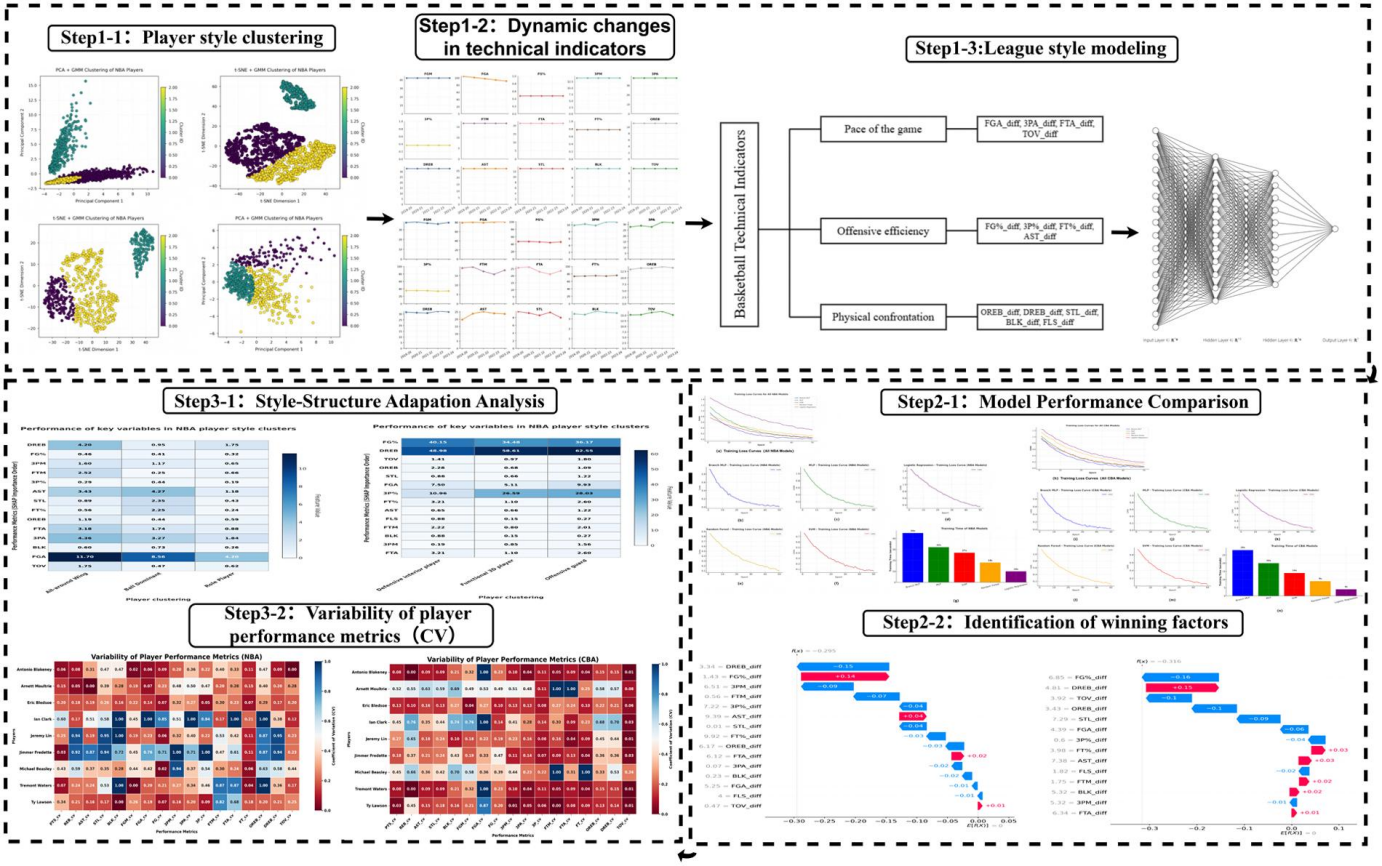
Technical workflow of the methodology.

Traditional research typically attributes underperformance of cross-league players to external environmental factors such as league policies, training systems, and individual capability gaps. Earlier work pointed out that the CBA's strict transfer rules and high development fees limit the international mobility and career growth of outstanding players (6); the misalignment between domestic youth development systems and international standards likewise leaves some players at a disadvantage in physical confrontation and tactical execution (7, 32). Moreover, objective differences in conditioning, explosiveness, technical detail, and game experience have long been regarded as major constraints on CBA players' ability to gain a lasting foothold in the NBA (8–10, 33, 34). However, these studies primarily focus on institutional, training, and physical factors while overlooking the fit between a player's personal style and the tactical systems and role distributions of different leagues. Meanwhile, research has shown that relying solely on static technical statistics makes it difficult to analyze the dynamic interaction between players' in-game behaviors and league styles (11–13). These two gaps leave scholars with a still-fragmentary understanding of cross-league performance adaptation, and the scarcity of studies on this issue limits theoretical innovation and the development of targeted practical strategies. Therefore, a dynamic perspective is needed to explore the matching mechanism between player styles and league tactical preferences, so as to identify the core factors influencing athletes' performance across leagues. Figure 1 shows the technology roadmap.

### 1.1 Literature review

Early analyses of basketball performance largely relied on structured technical statistics—such as field-goal percentage, rebounds, and turnovers—to explain player performance, key determinants of game outcomes, and team tactics. For example, Gómez et al. (14) showed that core technical statistics like shooting accuracy, rebounding, and turnovers effectively distinguished winning from losing teams in Spain's men's professional basketball league (14). Kubatko et al. (15), in a systematic review of statistical methods used in the NBA and other professional leagues, identified shooting, rebounding, and turnovers as the “starting point” for assessing player performance and team tactical execution (15). Sampaio and Janeira (16) used possession-based and other structured metrics to analyze the determinants of victory and stressed the central role of these statistics in understanding game outcomes and team performance (16). Although crucial in early performance studies, such indicators remain limited in revealing the deeper mechanisms underlying wins and losses.

With advances in data analysis, artificial intelligence, and player-tracking technologies, basketball analytics has shifted from traditional structured statistics to highly intelligent and automated methods. Static technical numbers alone cannot capture the complexity of tactics and decision-making processes, prompting researchers to adopt machine-learning approaches to mine temporal features and deeper structures. Li (17) used unsupervised clustering and temporal modeling of player-tracking data to automate the identification and analysis of defensive strategies (17). Lin et al. (18) proposed a spatiotemporal deep-learning framework that employs LSTM models to extract temporal tactical features from multisource data (18). Sukumaran et al. (19) noted that algorithms such as K-means, spectral clustering, DBSCAN, LSTM, and hidden Markov models have become core tools for modern tactical recognition and game-outcome prediction (19). However, despite their strong predictive power, these “black-box” models lack transparency, making it hard to explain how key variables influence performance. Lundberg and Lee's SHAP (SHapley Additive exPlanations) method (2017) offers a unified, theoretically grounded interpretability framework that quantifies each feature's contribution to a prediction (20). Consequently, interpretable machine-learning techniques have become a research hotspot, shifting the focus from “accurate prediction” to “transparent explanation”.

SHAP and related interpretability algorithms are now widely applied in basketball analytics, yet most studies concentrate on ranking variable importance or providing *post-hoc* explanations of outcomes (18, 21, 22). Few have developed forward-looking models that systematically analyze the “adaptation mechanism” linking player styles to league tactical structures. As a result, explanation frameworks based solely on global feature importance fail to reveal the complex, dynamic pathways through which multiple factors interact in basketball.

The multilayer perceptron (MLP), a classic deep-learning model, excels at feature extraction and nonlinear mapping but is often constrained to a single pathway when handling tasks involving numerous, structurally complex factors. This hampers its ability to capture hierarchies and multipath interactions among variables (23, 24). The branch-type multilayer perceptron (Branch-MLP) addresses this limitation by assigning separate branches to different key factors, enabling hierarchical modeling that systematically reveals the adaptation mechanisms between player styles and league tactical structures. Branch-MLP has enhanced the modeling of complex relationships in fields such as multitask recommendation systems (25) and multimodal sentiment analysis (26) but has yet to be applied to sports analytics. In basketball, Branch-MLP can allocate distinct branches to player styles, tactical features, and game-environment variables, hierarchically extracting and fusing diverse factors to better uncover how players and teams adapt within evolving tactical systems.

**Figure 2:**
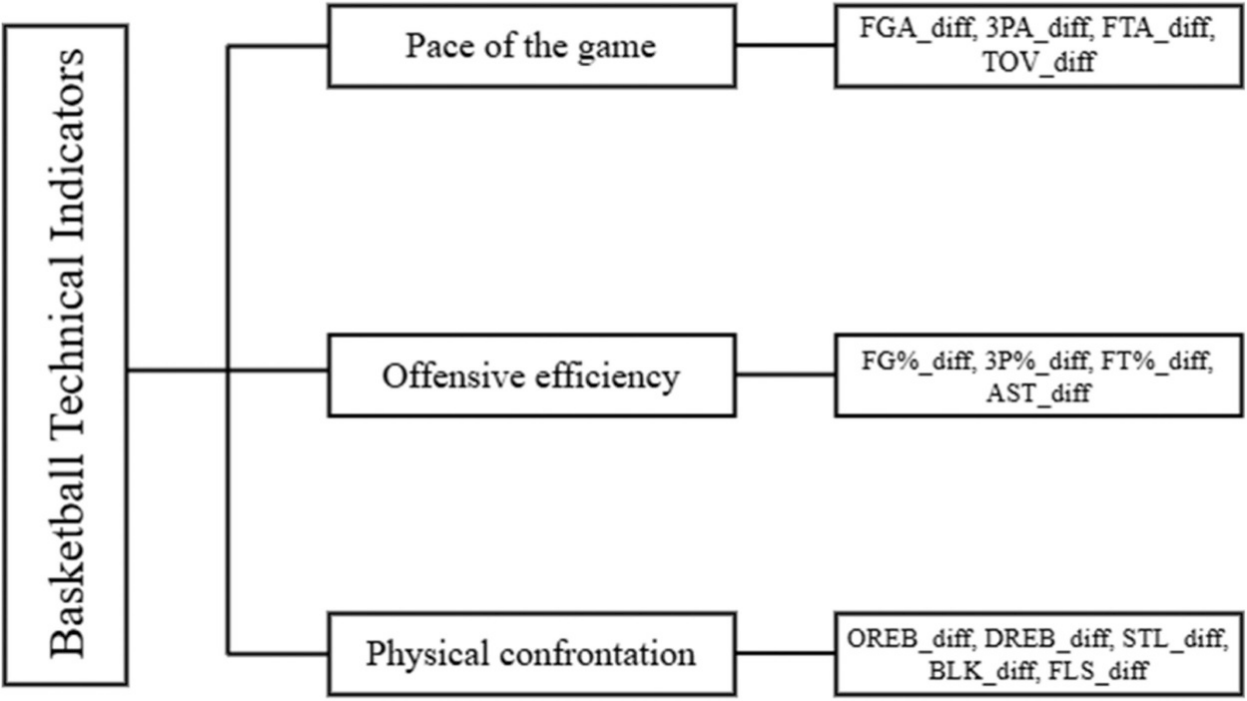
Classification framework of basketball technical indicators.

Building on prior work, this study constructs a two-level analytical framework to explore the fit between player game styles and league tactical structures and to quantify its impact on cross-league performance. At the player level, PCA and t-SNE dimensionality-reduction methods are combined with Gaussian mixture models to cluster season-long technical statistics and identify latent style types. At the league level, traditional technical metrics form the basis of an index system covering pace, offensive execution, and physical confrontation; a Branch-MLP, together with XGBoost and SHAP values, then provides interpretable modeling of the ecological structure. The study seeks to answer two questions: (a) How do systematic tactical-structure differences between the NBA and CBA affect players' cross-league performance? (b) Do stylistic traits developed in a player's original league exhibit structural mismatches with the tactical environment of the target league, thereby influencing performance? By integrating clustering methods with deep-network interpretability, this work offers a new pathway for analyzing cross-league performance and supplies theoretical and practical tools for talent selection and youth-development strategies. Figure 2 shows the technology classification framework.

## 2 Materials and Methods

### 2.1 Data and sample

This study draws on regular-season data from the 2019 to 2024 NBA and CBA seasons (sourced from NBA Stats and the official CBA website). In total, statistics were collected for roughly 15,000 NBA games and 4,141 CBA games, covering both team-level and player-level metrics.

A player-season record was included if the athlete (i) appeared in at least 20 games during the season, (ii) averaged no fewer than 10 min per game, and (iii) had complete core statistics such as points, rebounds, assists, and steals. Seasons were excluded when key data were missing—for example, owing to injury, retirement, or other gaps. Ultimately, 2,770 “player-season” samples were retained (340–427 per NBA season and 140–160 per CBA season).

For cross-league cases, the focus was on players who transferred from the NBA to the CBA between the 2019 and 2024 seasons. After verification with Statmuse and the CBA website, nine players met the criteria, each with at least two complete seasons in both leagues. No CBA-to-NBA transfers were included, as the sample was too small and the data incomplete for analysis.

### 2.2 Player style and data standardization

To make player performance comparable across leagues and seasons, all core statistics (e.g., points, assists, rebounds, shooting percentages) are first converted to a per-36 min basis (Per-36 Min). Next, z-score standardization is applied to these metrics to minimize the effects of playing time, game pace, and season-to-season variation.

$$X_{\mathrm{per}36}=\frac{X_{\mathrm{raw}}}{\mathrm{Minutes}\;\mathrm{Played}}\times 36$$

To further eliminate distributional differences across seasons and leagues, all variables were normalized using the z-score:

$$Z_{i}=\frac{X_{i}-\mu }{\sigma }$$

### 2.3 League-level modeling method

To reduce the impact of opponent strength and schedule variability, this study constructs differential features for each game by calculating the difference in technical statistics between the home and away teams. For the j technical indicator in game k, the feature is defined as:

$$d_{\mathrm{kj}}=x_{\mathrm{kj}}^{\mathrm{hom}e}-x_{\mathrm{kj}}^{\mathrm{away}}$$

To accurately capture the heterogeneity of different categories of technical metrics within the tactical system and their nonlinear interactions, this study introduces a Branched Multilayer Perceptron (Branched-MLP) network for modeling. The design concept is as follows: input variables are divided into several independent sub-channels based on their tactical functions, and each category is fed into a separate MLP branch to learn the nonlinear mapping between that category and game outcomes. The hidden-layer outputs of all branches are then fused in a trunk network, enabling joint representation of multidimensional features and high-level information integration. The network structure is as follows:

$$h^{\left(i\right)}=\sigma (W^{\left(i\right)}x^{\left(i\right)}+b^{\left(i\right)}),i\in \{\;1,2,3\}\;$$

The final fused output is:

$$\hat{y}=\mathrm{Sigmoid}\;(W_{f}[h^{\left(1\right)},h^{\left(2\right)},h^{\left(3\right)}]+b_{f})$$

The loss function adopted is binary cross-entropy:

$$L=\frac{1}{N}\sum_{i=1}^{N}\left[y_{i}\log{\hat{y}}_{i}+(1-y_{i})\log(1-{\hat{y}}_{i})\right]$$

where N is the number of samples, yi is the true label, and ${\hat{y}}_{i}$ is the predicted probability. All model evaluation metrics include Accuracy, AUC, F1-score, and Log-loss. An 80/20 train-test split and five-fold cross-validation are employed for performance assessment.

### 2.4 Interpretability analysis

To enhance model interpretability, SHAP (SHapley Additive exPlanations) is employed to assess the importance of input variables. Given the high computational cost of calculating SHAP values directly on deep networks, this study adopts a surrogate modeling approach (20), in which an XGBoost model is trained to approximate the output of the primary model. The mapping is defined as follows:

$${\hat{y}}_{\mathrm{MLP}}=\mathrm{MLP}(x),{\hat{y}}_{\mathrm{XGB}}=\mathrm{XGB}(x)$$

The overall importance of each ecological dimension is obtained by summing the SHAP values of all variables within that dimension:

$$\mathrm{SHA}P_{\dim\text{-}1}=\sum_{j\in V_{i}-}\mathrm{SHA}P_{j}$$

Here, $V_{i}$ denotes the complete set of features belonging to the i ecological dimension. All analyses were conducted in a Python 3.10 environment, primarily using the scikit-learn, XGBoost, SHAP, and PyTorch libraries. Data and code are available upon request. This study does not involve human or animal subjects and therefore does not require ethical review.

## 3 Results

### 3.1 Clustering structure of player styles

**Figure 3:**
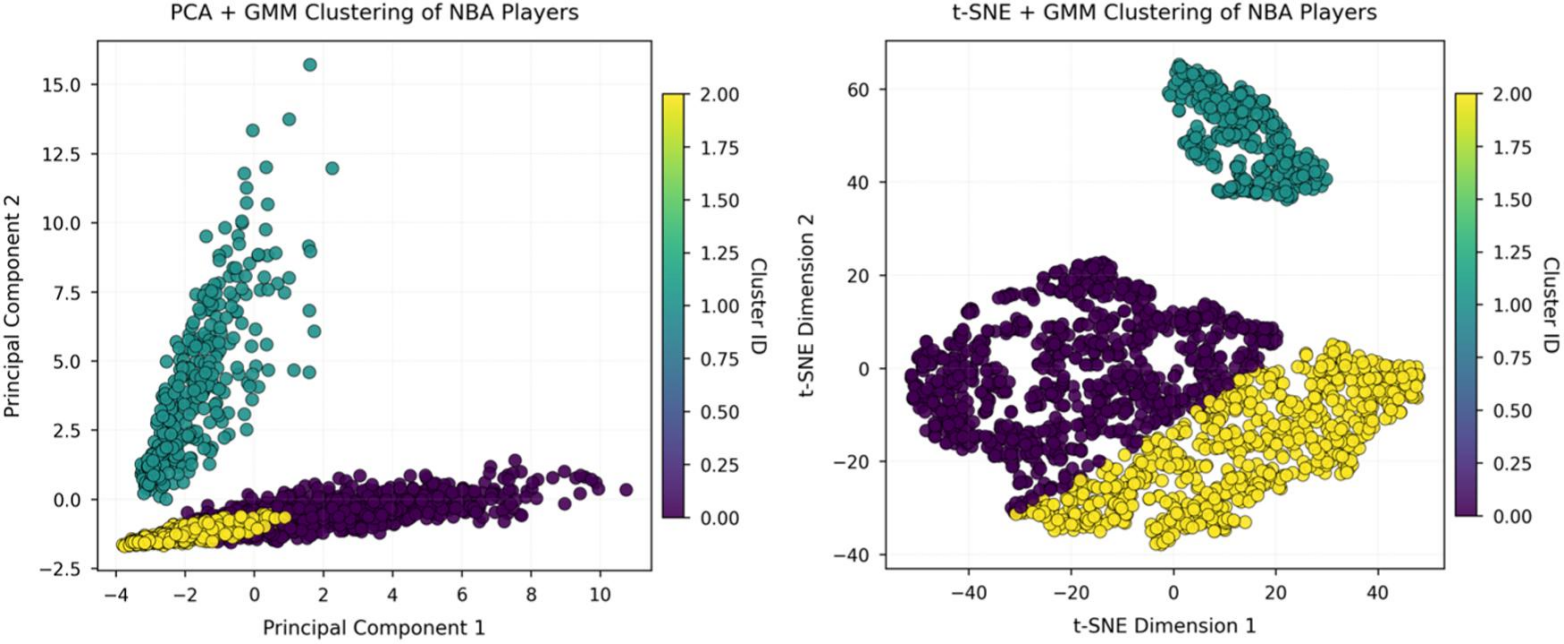
Visualization of NBA player-style clusters under two dimensionality-reduction methods.

**Figure 4:**
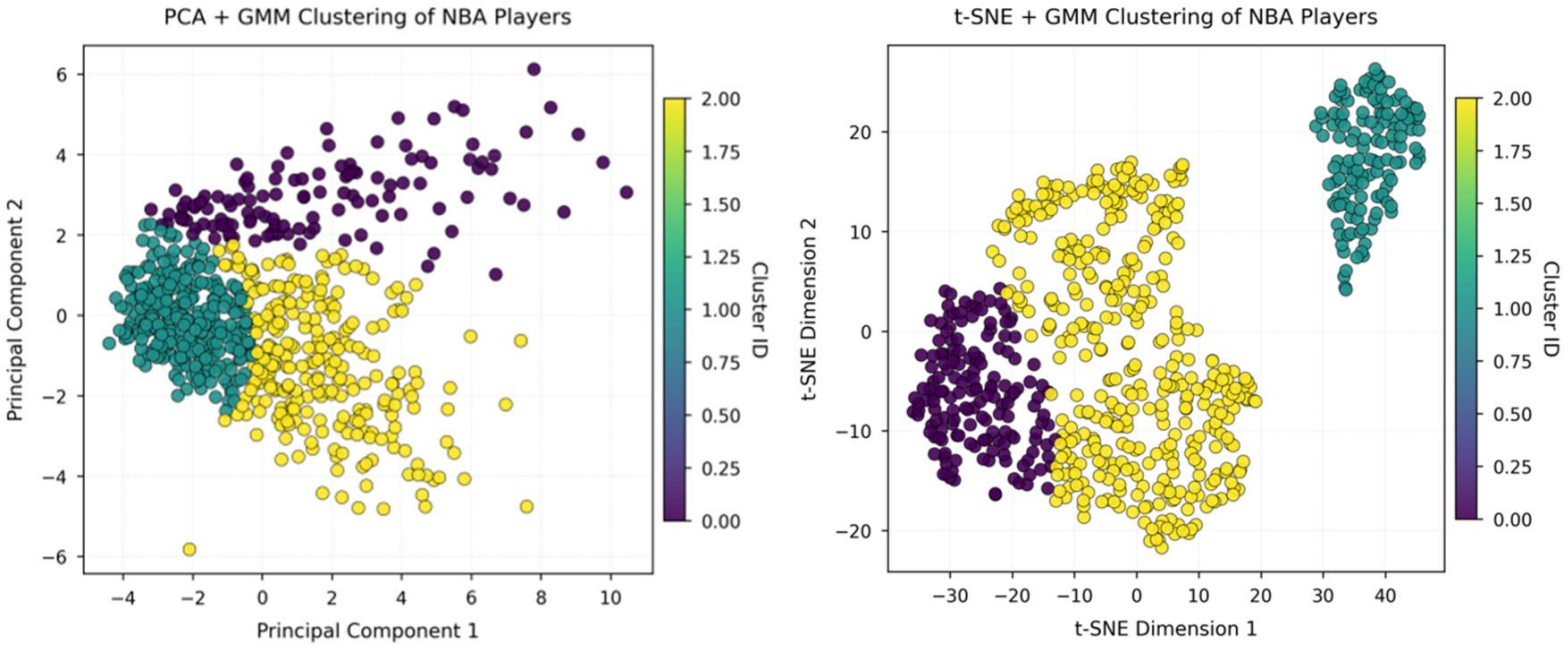
Visualization of CBA player-style clusters under two dimensionality-reduction methods.

After performing PCA and t-SNE dimensionality reduction on NBA and CBA player statistics, an unsupervised clustering of player styles was completed with a Gaussian mixture model (GMM). Figures 3, 4 display the clustering distributions of players from the two leagues in the respective low-dimensional spaces; different colors indicate the style types identified by the GMM.

Figures 3a,b show the NBA results in the t-SNE and PCA spaces, respectively. In the t-SNE projection, the boundaries between style groups are clearer and the structural differentiation is higher, indicating that the nonlinear method captures the complex relationships among style variables more effectively. In the PCA space, by contrast, the clusters are more compact and some boundaries overlap, suggesting that linear dimensionality reduction has certain limitations in distinguishing NBA player styles.

Figures 4c,d present the clustering structure for CBA players under the two projections. Overall, the CBA clusters are relatively compact in both the t-SNE and PCA spaces, with some overlap between groups. This reflects a more concentrated style ecology in the CBA and less pronounced group differentiation than in the NBA.

In sum, t-SNE more effectively reveals the intrinsic structure of player styles than PCA, and both the diversity and differentiation of NBA player styles exceed those of the CBA—underscoring fundamental league-level differences in player development and role allocation.

#### 3.1.1 Comparison of NBA and CBA player-style clusters

To further compare the stylistic structures of NBA and CBA players, the study divides players in each league into three typical style clusters on the basis of the GMM results (see Tables 1, 2) and quantifies their main technical indicators. The clustering reveals that although both leagues contain versatile, offense-oriented, and role-oriented player categories, the specific skill profiles and functional distributions of these groups differ markedly.

**Table 1:** Mean technical profile of NBA player-style clusters.

Cluster ID	FGM	FGA	FG%	3PM	3PA	3P%	FTM	FTA	FT%	OREB	DREB	TRB	AST	STL	BLK	TOV	Style name
Cluster 0	5.568	11.696	0.457	1.603	4.362	0.286	2.520	3.179	0.560	1.188	4.195	5.383	3.431	0.892	0.598	1.753	All-Around Wing
Cluster 1	23.017	3.952	8.563	0.411	1.173	3.267	0.252	1.742	2.249	0.438	0.954	3.313	4.270	2.349	0.731	0.466	Ball-Dominant Core
Cluster 2	1.901	4.199	0.318	0.648	1.843	0.190	0.659	0.880	0.239	0.592	1.748	2.339	1.175	0.432	0.255	0.623	Role Player

**Table 2:** Mean technical profile of CBA player-style clusters.

Cluster ID	FGM	FGA	FG%	3PM	3PA	3P%	FTM	FT%	OREB	DREB	TRB	AST	STL	BLK	TOV	Style nam
Cluster 0	4.158	7.502	40.152	0.190	0.588	10.955	2.220	3.209	48.985	2.279	4.207	6.479	0.648	0.876	1.408	Defensive Interior Player
Cluster 1	2.185	5.108	34.475	0.850	2.443	26.585	0.802	1.100	58.614	0.680	1.681	2.362	0.658	0.150	0.973	3D Specialist
Cluster 2	4.412	9.930	36.168	1.558	4.461	28.034	2.013	2.595	62.554	1.089	3.006	4.093	1.220	0.273	1.802	Offensive Guard

For NBA players, Versatile Wings (Cluster 0) exhibit a balanced blend of scoring, defense, and playmaking, with average points, rebounds, and assists all at moderate-to-high levels. This suggests that such players serve as multipurpose hubs in transition at both ends of the floor. On-Ball Scorers (Cluster 1) are distinguished by offense dominance: their FGA (23.02), AST (4.27), and TOV (2.35) are the highest of the three clusters, indicating they initiate most of the team's offense and enjoy greater ball-handling and decision-making opportunities. By contrast, Role Players (Cluster 2) record the lowest means in core metrics such as scoring and assists, emphasizing defense, rotation duties, and specific tactical complements; this reflects the NBA's fine-grained role specialization.

The CBA clusters show a different distribution. Defensive Bigs (Cluster 0) post the highest numbers in defensive metrics such as rebounds (TRB = 6.48) and blocks (BLK = 0.88), underscoring their rim-protection and help-defense roles. 3-and-D Specialists (Cluster 1) combine a degree of three-point shooting (3PM = 0.85) with defensive contributions (STL = 0.66); although their shooting percentages are not high, they are tasked with spacing the floor and rotating defensively. Offensive Guards (Cluster 2) excel in scoring-related metrics—FGA (9.93), FGM (4.41), AST (1.22)—indicating heavier responsibility for scoring and back-court orchestration.

In sum, both leagues display stylistic diversity, but NBA clusters show sharper differentiation and clearer functional separation, reflecting greater tactical variety and specialization. CBA clusters are more compact with partial functional overlap, pointing to a more concentrated style ecosystem and a convergent tactical system.

### 3.2 Dynamic evolution of team tactical structures

Building on the static player-style findings, this section analyzes the year-to-year evolution of NBA and CBA team tactics—using annual core technical indicators—and compares the two leagues.

**Figure 5:**
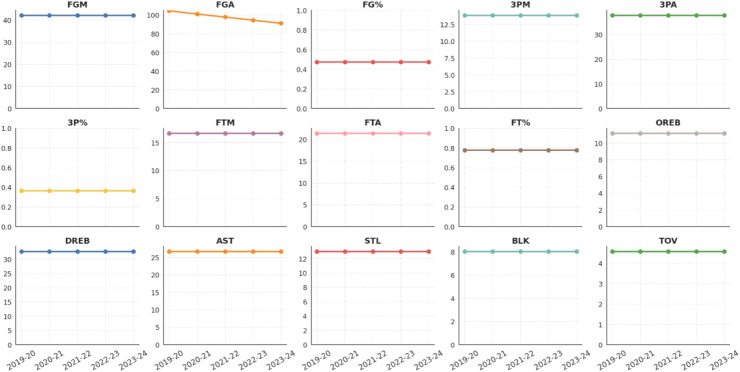
Annual evolution of core technical indicators for NBA teams.

**Figure 6:**
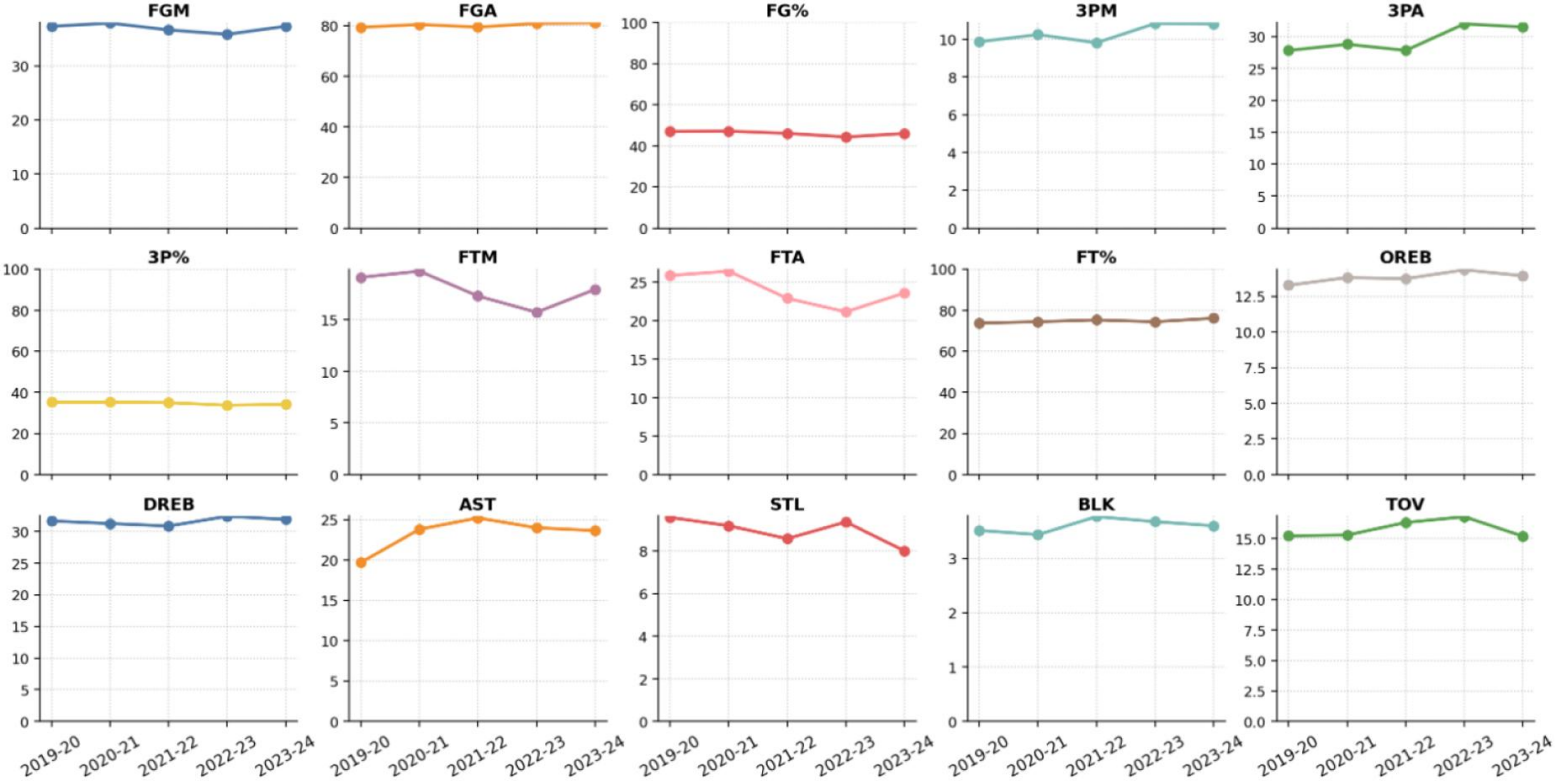
Annual evolution of core technical indicators for CBA teams.

Figures 5, 6 trace trends in key offensive, shooting, and defensive metrics for NBA and CBA teams from the 2019–2024 seasons. A time-series view of these indicators reveals fundamental differences in each league's tactical evolution and structural stability.

#### 3.2.1 Offensive efficiency and shooting structure

Across the past five seasons, NBA teams’ field goals made (FGM), field-goal attempts (FGA), field-goal percentage (FG%), and three-point metrics (3PM, 3PA, 3P%) have remained essentially flat, with only minimal year-on-year variation. Team-play indicators such as assists (AST) are equally stable, indicating a mature, highly codified modern-basketball system. Core concepts—floor spacing, perimeter creation, and pick-and-roll initiation—have been fully internalized league-wide, and offensive roles are finely specialized. This stability underpins both efficient scoring and the league's ongoing emphasis on high-level tempo control and transition play.

#### 3.2.2 Defense and transition

Defensive and transition statistics (steals STL, blocks BLK, offensive and defensive rebounds OREB/DREB) exhibit the same high stability. This reflects sustained investment in defensive schemes and the league's stringent demands on two-way balance and role clarity. Overall, the NBA's tactical ecosystem is highly organized and steady, with core metrics showing negligible inter-season fluctuations.

By contrast, CBA teams demonstrate far more pronounced year-to-year swings in the same indicators. Offensive metrics—FG%, 3PA, 3PM, free throws, and AST—fluctuate noticeably more than in the NBA. Some seasons show temporary upticks in three-point volume and assists, suggesting that the CBA is actively absorbing modern concepts to enhance spacing and team play; yet the overall rise in perimeter efficiency remains limited, indicating challenges in fully integrating new offensive structures. Defensive figures such as steals and blocks also vary from season to season, highlighting room for improvement in scheme stability and execution.

In short, the NBA's core metrics are remarkably steady year over year, reflecting a highly professionalized league with a deeply entrenched tactical system. The CBA, by contrast, displays larger metric swings and frequent stylistic adjustments, indicating that the league as a whole is still in a phase of rapid evolution and conceptual renewal. These structural differences underscore fundamental contrasts in tactical style, ecological maturity, and organizational sophistication between the two competitions.

Validate the applicability and robustness of the branched modeling approach in capturing complex patterns within heterogeneous ecological systems.

To comprehensively assess the training efficiency and convergence behavior of different models on NBA and CBA league data, Figure 4 and Figure 5 present the training loss curves and training time comparisons for each dataset, respectively. Figure 4 shows results obtained from the NBA dataset, while Figure 5 displays corresponding outcomes from the CBA dataset. Each figure includes five models—Branched-MLP, MLP, SVM, Random Forest, and Logistic Regression—offering a detailed visualization of model convergence trajectories and computational cost.

These comparative plots not only reveal performance differences in training dynamics but also provide insights into how league-specific ecological structures influence model learning behavior and feature extraction capacity across different data environments.

As shown in Figure 4, on the NBA dataset, Branch-MLP and MLP exhibit faster loss convergence and lower final loss values, while SVM and Random Forest (RF) converge more slowly. Figure 5 indicates that on the CBA dataset, the performance gap among models narrows, though Branch-MLP still maintains a certain advantage.

In terms of training time, both datasets reveal that deep learning models incur higher computational costs compared to traditional models, but offer superior capabilities in feature extraction and pattern recognition. This comparison underscores the significant influence of league-level ecological structures on model learning difficulty and performance variation.

#### 3.2.3 Tactical-style clustering and type distribution

Building on the analysis of year-to-year changes, this section uses season-level core metrics to perform unsupervised Gaussian-mixture clustering of NBA and CBA teams, allowing a comparison of each league's internal tactical structure.

##### 3.2.3.1 NBA clusters

High-Efficiency Synergy (Cluster 0) posts the league's highest assists (AST = 27.64) and three-point makes (3PM = 14.53), with a leading field-goal percentage (FG% = 0.485), indicating superior offensive efficiency and coordination.Pressure-Defense/Fast-Break (Cluster 1) records high steals (STL = 13.52) and personal fouls (FLS = 20.2) while keeping turnovers (TOV = 4.16) the lowest of the three clusters, highlighting disruptive defense and strong transition play.

Balanced All-Round (Cluster 2) shows mid-range values across the board—field goals made (FGM = 41.11), assists (AST = 25.59), steals (STL = 13.08), etc.—with no glaring weaknesses.

##### 3.2.3.2 CBA clusters

Traditional Half-Court Offense (Cluster 0) posts moderate figures in offensive rebounds (OREB = 13.37), assists (AST = 23.25), and three-point makes (3PM = 9.68), reflecting a steady, set-play style.High-Efficiency Transition Offense (Cluster 1) leads the three clusters in OREB = 14.22, 3PM = 10.68, and FG% = 0.467, with slightly higher assists (AST = 23.61), indicating strong efficiency and transition capability. Interior-Defense Balanced (Cluster 2) excels in defensive rebounds (DREB = 31.43) and blocks (BLK = 3.71) while maintaining mid-range offensive numbers, yielding a balanced attack–defense profile.

Overall, both leagues exhibit distinct tactical types, with each cluster showing characteristic values in organization, perimeter shooting, defense, and rebounding. Detailed statistics are provided in Tables 3, 4.

**Table 3:** Mean values of cluster characteristics for NBA teams.

Cluster ID	OREB	DREB	AST	STL	BLK	TOV	FLS	FGM	FGA	FG%	3PM	3PA	3P%	FTM	FTA	FT%	Style nam
Cluster 0	11.01	33.7	27.64	12.57	8.19	4.83	18.8	43.41	107.05	0.485	14.53	38.53	0.377	17.21	22.05	0.779	High-Efficiency Synergy
Cluster 1	11.32	31.77	25.96	13.52	7.85	4.16	20.2	40.84	88.93	0.461	13.18	37.09	0.355	15.72	20.33	0.774	Pressure-Defense/Transition Fast-Break
Cluster 2	11.11	32.08	25.59	13.08	8.05	4.86	19.4	41.11	89.13	0.463	13.2	37.25	0.354	17.01	21.92	0.773	Balanced All-Round

**Table 4:** Mean values of cluster characteristics for NBA teams.

Cluster ID	OREB	DREB	AST	STL	BLK	TOV	FLS	FGM	FGA	FG%	3PM	3PA	3P%	FTM	FTA	FT%	Style nam
Cluster 0	13.37	31.08	23.25	8.28	3.47	16.31	23.67	35.84	78.91	0.454	9.68	28.59	0.338	17.25	23.14	0.744	Traditional Half-Court Offense
Cluster 1	14.22	32.08	23.61	9.34	3.64	15.42	23.38	37.97	81.3	0.467	10.68	30.15	0.353	18.57	24.52	0.757	High-Efficiency Transition Offense
Cluster 2	13.7	31.43	22.75	9.08	3.71	15.72	23.59	36.8	80.56	0.457	10.54	30.05	0.349	17.62	23.96	0.736	Interior-Defense Balanced

### 3.3 League-style modeling results

#### 3.3.1 Model-prediction outcomes and performance comparison

This section presents the game-outcome (win/loss) prediction performance of different models on the NBA and CBA data sets. Full model structures and training details are described in § 2.3. Five mainstream classifiers were compared: Branched-MLP, standard MLP, support-vector machine (SVM), random forest (RF), and logistic regression (LR). Hyper-parameters were optimized by grid search, and five-fold cross-validation was used to assess generalization. Precision, Recall, F1-score, AUC, and Accuracy are reported. Tables 3, 4 list the mean cross-validated results for the NBA and CBA sets, respectively.

##### 3.3.1.1 NBA results

Branched-MLP performed best, achieving an F1-score of 0.9813, a 2.37% improvement over MLP (0.9576). Its AUC reached 0.9973, 2.23% higher than LR, and its Accuracy was 0.9814, nearly six percentage points above SVM. These gains show that the branched architecture captures ecological structure differences and key win-loss features in NBA games more effectively.

##### 3.3.1.2 CBA results

Performance gaps narrowed markedly. Branched-MLP's F1-score was 0.8844, only 0.01%–0.02% above MLP (0.8843) and SVM (0.8842). Its AUC was 0.9541, 1.79% higher than LR (0.9362). Although its structural advantage is less pronounced than in the NBA set, Branched-MLP still leads on Precision, Recall, and Accuracy.

##### 3.3.1.3 Overall

Branched-MLP shows stronger feature-learning and pattern-recognition capabilities in the NBA's more stable tactical environment with clearer player-role stratification. In the CBA's looser structure, model performances converge, yet Branched-MLP maintains a 1%–2% edge, confirming the suitability and robustness of the branched design in complex, highly heterogeneous contexts.

#### 3.3.2 Comparison of model convergence and training efficiency

**Figure 7:**
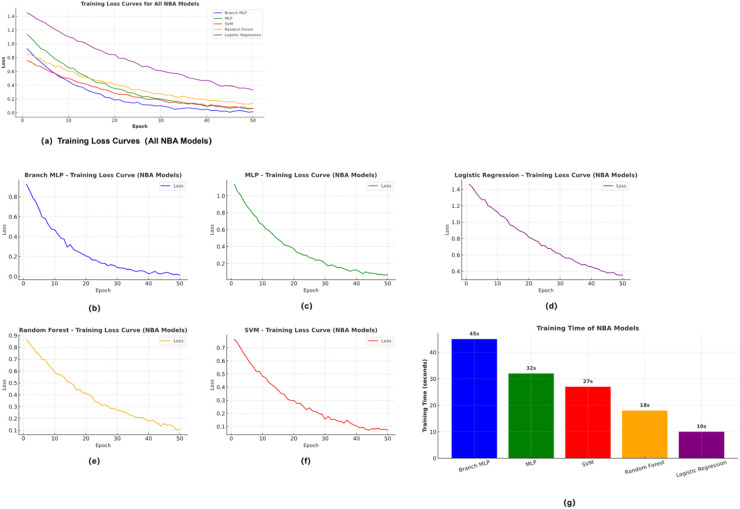
Training loss and efficiency on NBA dataset.

**Figure 8:**
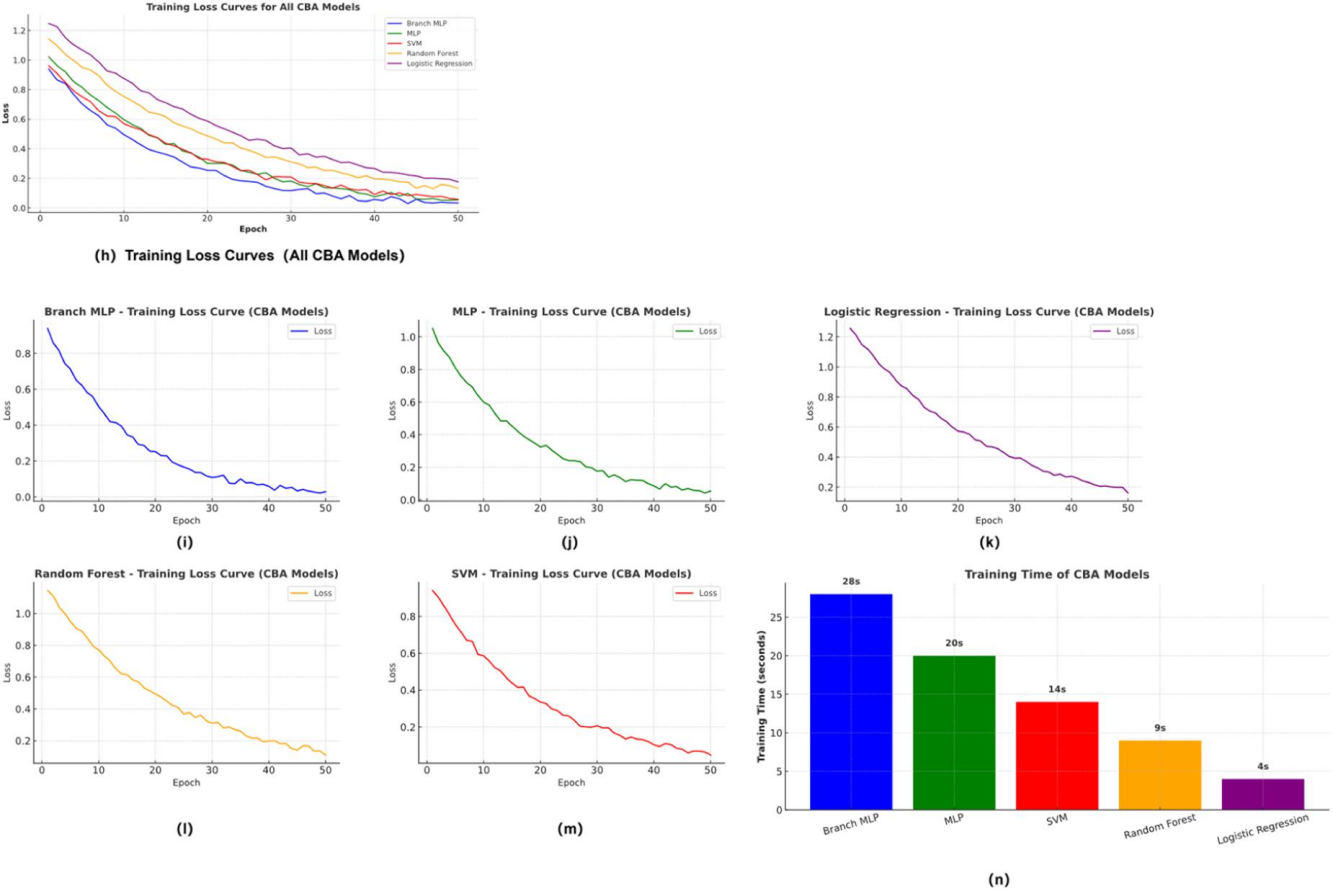
Training loss and efficiency on CBA dataset.

To further illustrate the training efficiency and convergence behavior of the various models on NBA and CBA data, Figures 7, 8 present each dataset's training-loss curves and training-time comparisons. Figure 7 shows the NBA results, while Figure 8 corresponds to the CBA. Each figure includes five models—Branched-MLP, multilayer perceptron (MLP), support-vector machine (SVM), random forest (RF), and logistic regression (LR)—and contrasts their loss trajectories and computational cost during training. These visualizations intuitively reflect the models' convergence dynamics and training-efficiency differences in the two data environments.

As Figure 7 indicates, Branched-MLP and MLP achieve faster loss convergence and lower final loss values on the NBA dataset, whereas SVM and RF converge more slowly. Figure 8 shows that the performance gaps narrow on the CBA dataset, though Branched-MLP still retains a slight edge in convergence speed and final loss. Regarding training time, both datasets reveal that deep-learning models incur higher computational costs than traditional models, yet they deliver stronger feature-extraction and pattern-recognition capabilities. This comparison offers a clear view of the learning-efficiency and training-performance differences across models for the two datasets.

#### 3.3.3 Feature-importance and interpretability analysis

**Figure 9:**
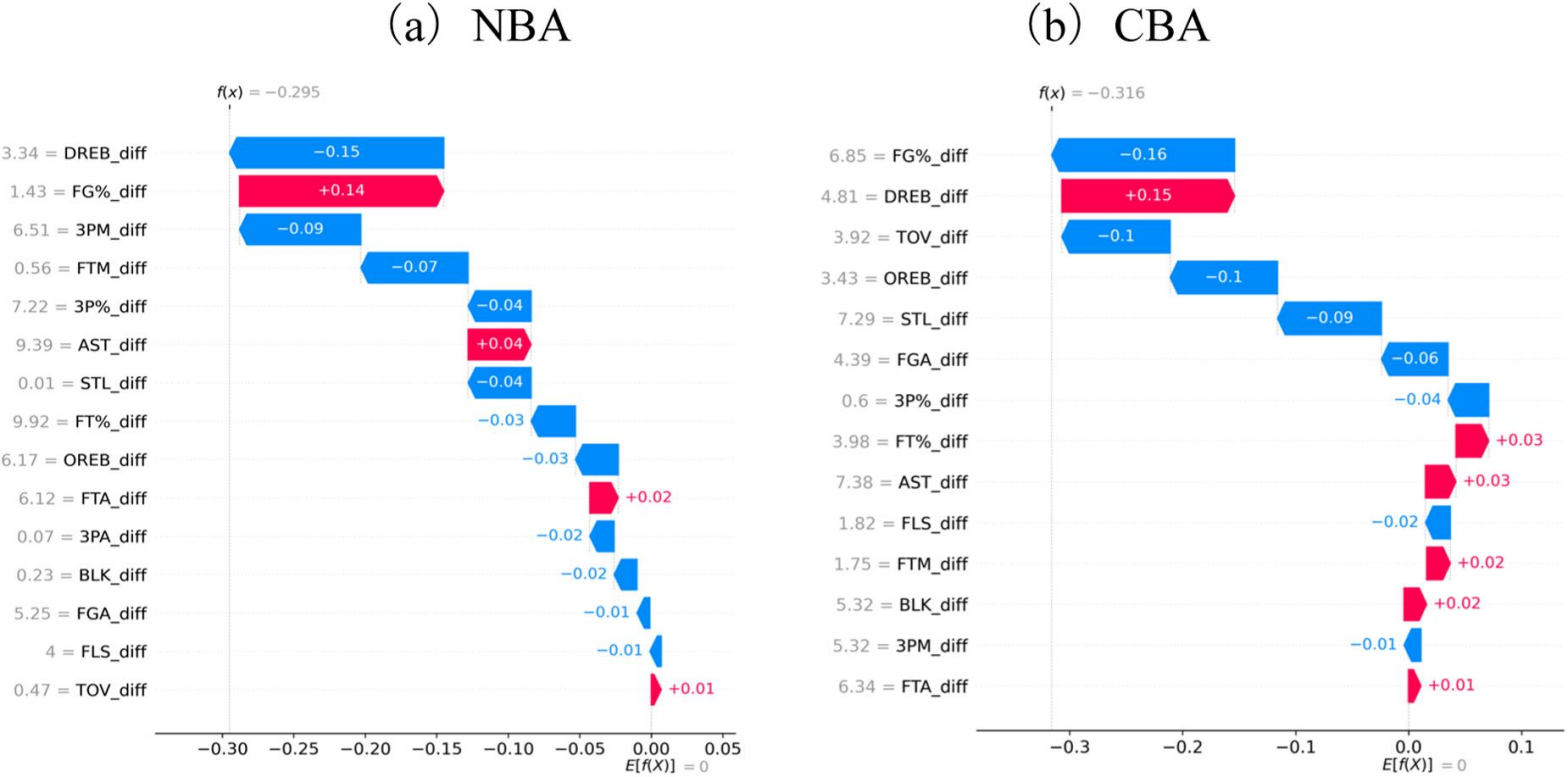
Importance ranking of ecological variables for win–loss prediction across leagues.

To enhance the interpretability of the deep-learning models, an XGBoost surrogate model was trained, and SHAP (SHapley Additive exPlanations) values were used to quantify how each technical statistic actually affects win-loss predictions. Figures 9a,b show bar charts ranking feature importance by SHAP values for the NBA and CBA datasets, respectively, while Tables 5, 6 provide the quantitative average contributions of each feature.

**Table 5:** Performance comparison of different models on the NBA dataset (mean of 5-fold cross-validation).

Algorithm	Precision	Recall	F1 Score	AUC	Accuracy
Branch MLP	0.9780	0.9847	0.9813	0.9973	0.9814
MLP	0.9605	0.9556	0.9576	0.9921	0.9575
SVM	0.9230	0.9197	0.9200	0.9714	0.9214
Random Forest	0.9509	0.9349	0.9428	0.9878	0.9417
Logistic Regression	0.9207	0.9103	0.9147	0.9751	0.9169

**Table 6:** Performance comparison of different models on the NBA dataset (mean of 5-fold cross-validation).

Algorithm	Precision	Recall	F1 Score	AUC	Accuracy
Branch MLP	0.8822	0.8900	0.8844	0.9541	0.8825
MLP	0.8811	0.8877	0.8843	0.9531	0.8821
SVM	0.8787	0.8862	0.8842	0.9528	0.8824
Random Forest	0.8619	0.8794	0.8706	0.9489	0.8676
Logistic Regression	0.8550	0.8599	0.8568	0.9362	0.8550

In both panels, the SHAP weights concentrate heavily among the top features, yet the specific rankings and influential factors differ noticeably between the two leagues.

##### 3.3.3.1 NBA dataset

Defensive rebounds (DREB_diff, 0.150 ± 0.084) and field-goal percentage (FG%_diff, 0.143 ± 0.060) are the two variables on which the model relies most. Defensive rebounds not only cut off opponents' second-chance opportunities but often initiate high-quality transition offense. Shooting percentage captures a team's overall scoring efficiency and finishing ability and is decisive under high-intensity competition.Three-point makes (3PM_diff, 0.085) and three-point percentage (3P%_diff, 0.044) rank close behind, underscoring how perimeter shooting has become critical for spacing, creating mismatches, and boosting scoring bursts in modern NBA play.Collaboration and defensive-to-offense transition metrics such as assists (AST_diff, 0.044) and steals (STL_diff, 0.044) also carry substantial weight, reflecting the league's fast pace and frequent end-to-end actions where teamwork and pressure defense are indispensable.Other features—free throws, offensive rebounds, fouls—contribute less, indicating that, in this elite environment, basic scoring efficiency and perimeter production are the core lines of separation between winning and losing (Figure 9a; Table 5). The complete SHAP-based ranking for all NBA features is presented in Table 7.

**Table 7:** SHAP-Based feature importance ranking for the NBA dataset.

Rank	Feature	SHAP Importance (Mean ± SD)
1	DREB_diff	0.150 ± 0.084
2	FG%_diff	0.143 ± 0.060
3	3PM_diff	0.085 ± 0.062
4	FTM_diff	0.074 ± 0.062
5	3P%_diff	0.044 ± 0.029
6	AST_diff	0.044 ± 0.027
7	STL_diff	0.044 ± 0.027
8	FT%_diff	0.031 ± 0.028
9	OREB_diff	0.030 ± 0.032
10	FTA_diff	0.020 ± 0.037
11	3PA_diff	0.017 ± 0.019
12	BLK_diff	0.016 ± 0.026
13	FGA_diff	0.009 ± 0.007
14	FLS_diff	0.008 ± 0.009
15	TOV_diff	0.007 ± 0.013

##### 3.3.3.2 CBA dataset

Field-goal percentage (FG%_diff, 0.162 ± 0.097) and defensive rebounds (DREB_diff, 0.153 ± 0.101) again top the list, but two variables rise sharply in importance: turnovers (TOV_diff, 0.096) and offensive rebounds (OREB_diff, 0.095) occupy the third and fourth positions. This shows that, in CBA games, ball-control (reducing turnovers) and rebounding battles weigh even more heavily on outcomes.Dependence on three-point production is relatively lower (3PM_diff = 0.015; 3P%_diff = 0.036), implying that the league still leans toward interior finishing and half-court attacks.Steals (STL_diff, 0.092) and interior-control indicators (rebounds at both ends, blocks) remain important on defense, but teamwork metrics such as assists carry less weight than in the NBA, suggesting differences in the maturity of spacing, ball distribution, and team scheme execution (Figure 9b; Table 6). A full SHAP-based ranking for the CBA dataset is provided in Table 8.

**Table 8:** SHAP-Based feature importance ranking for the CBA dataset.

Rank	Feature	SHAP Importance (Mean ± SD
1	FG%_diff	0.162 ± 0.097
2	DREB_diff	0.153 ± 0.101
3	TOV_diff	0.096 ± 0.073
4	OREB_diff	0.095 ± 0.069
5	STL_diff	0.092 ± 0.063
6	FGA_diff	0.059 ± 0.044
7	3P%_diff	0.036 ± 0.029
8	FT%_diff	0.029 ± 0.030
9	AST_diff	0.027 ± 0.023
10	FLS_diff	0.022 ± 0.021
11	FTM_diff	0.021 ± 0.017
12	BLK_diff	0.020 ± 0.018
13	3PM_diff	0.015 ± 0.014
14	FTA_diff	0.011 ± 0.012
15	FTA_diff	0.011 ± 0.011

##### 3.3.3.3 Joint observations

Shooting efficiency and defensive rebounding emerge as the shared top drivers of game results in both leagues. Yet the NBA puts greater emphasis on perimeter firepower and coordinated play, whereas the CBA highlights ball security and rebounding. The more concentrated SHAP distribution among top variables in the CBA may reflect a relatively narrower set of decisive factors in that league's style. Overall, the SHAP-based interpretability analysis sheds light on each league's tactical priorities and supplies data-backed guidance for improving model transparency and on-court strategy.

### 3.4 Alignment between player styles and league tactical structures

This section combines heat-map visualisations with coefficient-of-variation analysis to examine, in a systematic way, how player styles align with the mainstream tactical structures of each league. It then probes the season-to-season performance volatility of players who move between leagues.

**Figure 10:**
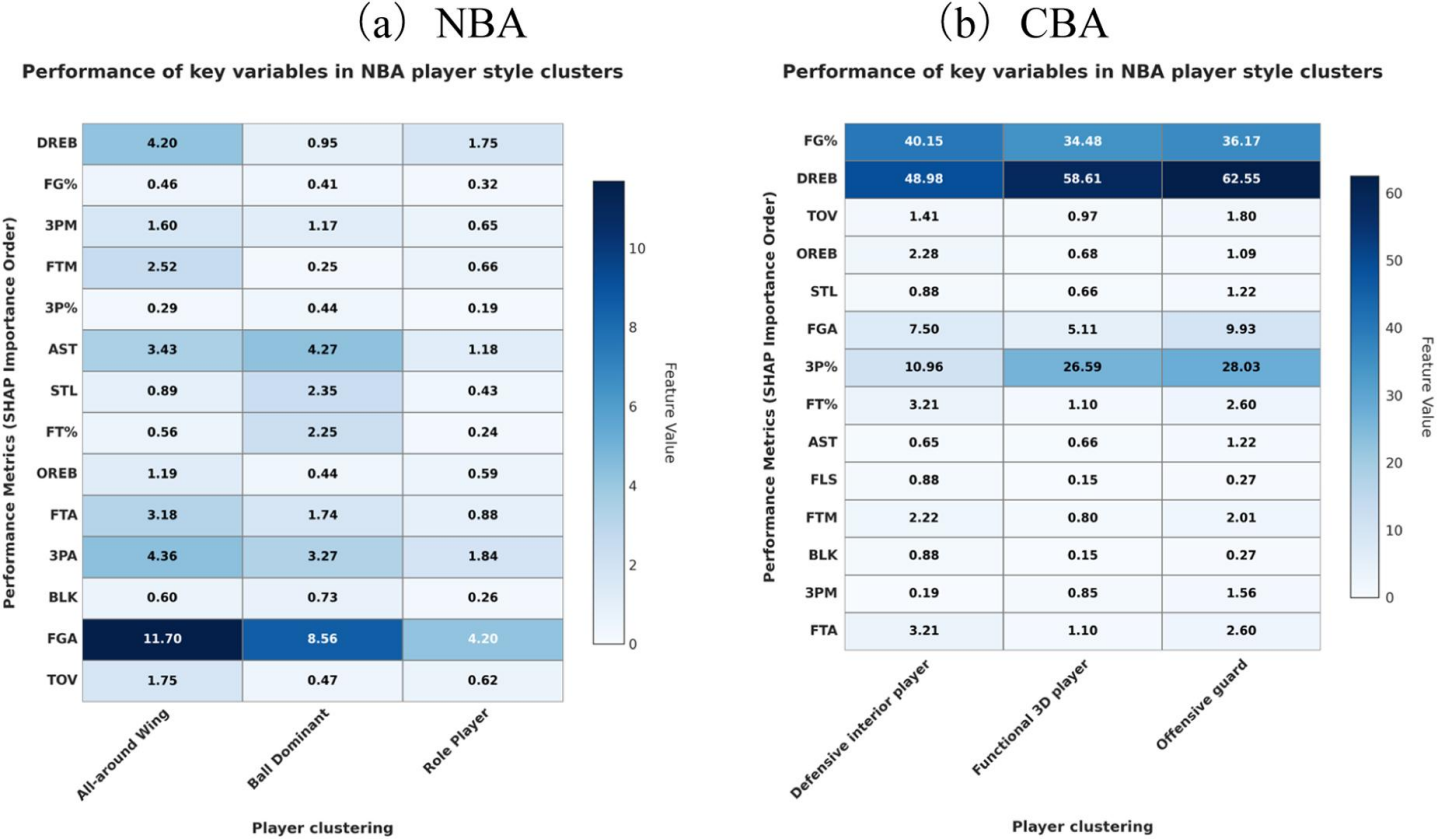
Comparison of key characteristics of NBA and CBA player style clusters.

Figures 10a,b) display the standardised mean scores of key technical indicators for every style cluster in the NBA and CBA, respectively, thus revealing each type's performance gradient on the core metrics.

#### 3.4.1 NBA clusters

All-Around Wing. Outstanding in assists, steals, and true-shooting percentage, this type blends offence, defence, and facilitation, serving as a critical hub in the NBA's spacing-focused, up-tempo ecosystem.Ball-Dominant Guard. Distinguished by high-usage metrics—shot attempts and points are the highest of all clusters, but turnovers also peak—mirroring the aggressive creation and risk-laden decision-making of a primary scorer. Role Player. Records the lowest means in most categories but maintains a middling three-point percentage, reflecting the NBA's finely segmented system in which specialists stretch the floor and execute defensive rotations.

#### 3.4.2 CBA clusters

Defensive Interior Player. Excels in defensive rebounds and blocks while keeping turnovers low, underlining its rim-protection and finishing duties.Functional 3-and-D Player. Defined by three-point volume, three-point accuracy, and steals, it anchors floor spacing and perimeter defence within the CBA context.Offensive Guard. Leads in shooting percentage, offensive rebounds, and free-throw makes, emphasising dribble penetration and foul-drawing ability.

#### 3.4.3 Cross-league contrasts

NBA style differentiation surfaces not only on offence and defence but also in organisation and spatial cooperation, with especially sharp gradients in perimeter firepower and team assists. CBA styles lean more toward rebounding battles, ball control, and interior finishing—echoing the league's tactical priorities. Noticeably, defensive rebounding and overall shooting efficiency are central metrics for interior clusters in both leagues, signalling the cross-league universality of these fundamental skills.

#### 3.4.4 Practical implications

Fit between player style and tactical structure affects individual output and directly shapes role assignment and adaptation difficulty when athletes switch leagues. Youth academies and professional training programmes should map development paths to the tactical demands of the target competition. For instance, guards aiming for the NBA should sharpen transition decision-making and cooperative reads, whereas bigs seeking success in the CBA should focus on physicality, rebounding, and finishing. Such tailored preparation can raise cross-system adaptation efficiency and support more stable, sustained performance trajectories.

#### 3.4.5 Style–tactics Fit and cross-league performance stability

**Figure 11:**
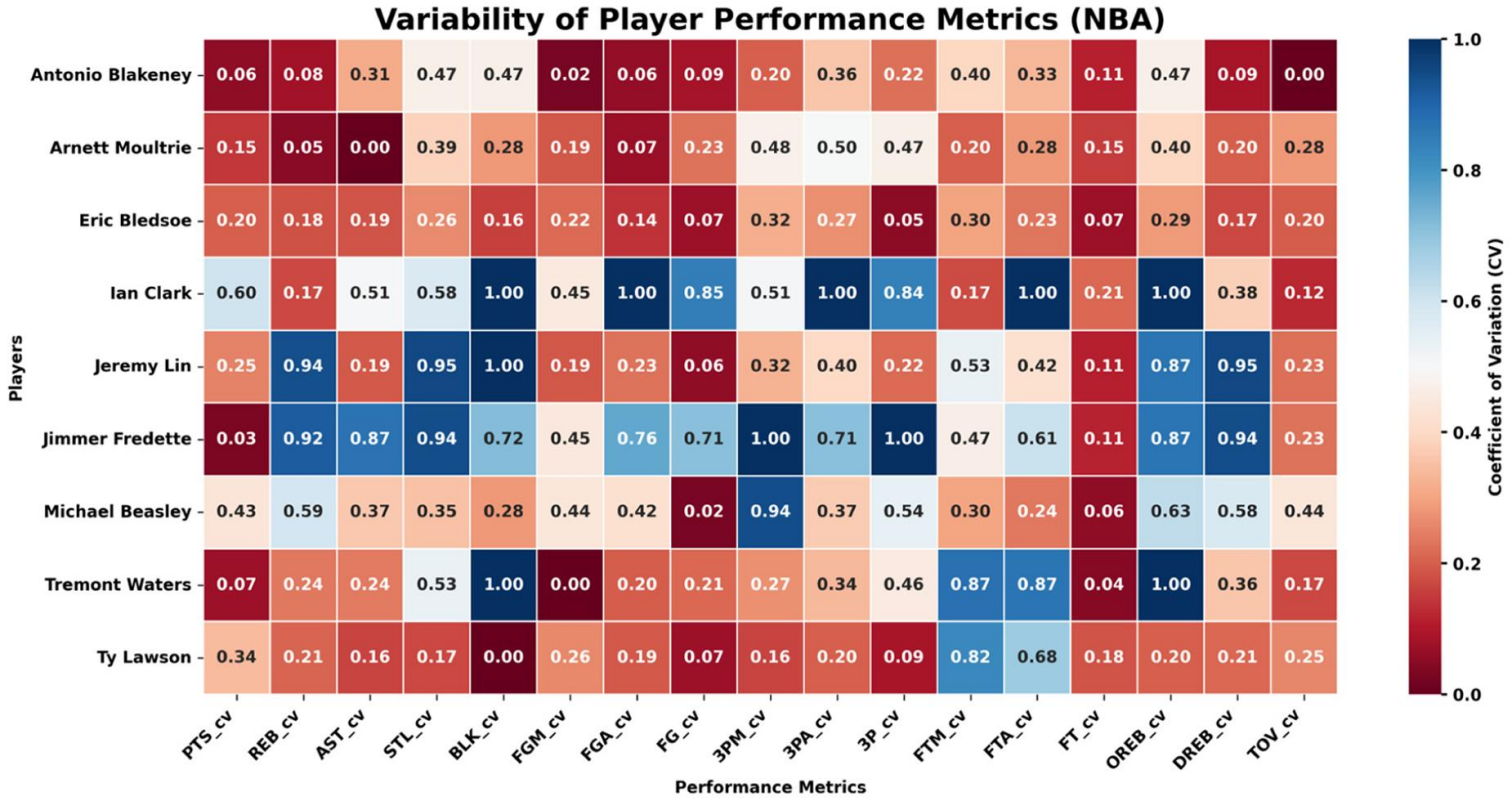
Variability of player performance metrics (CV) in NBA.

**Figure 12:**
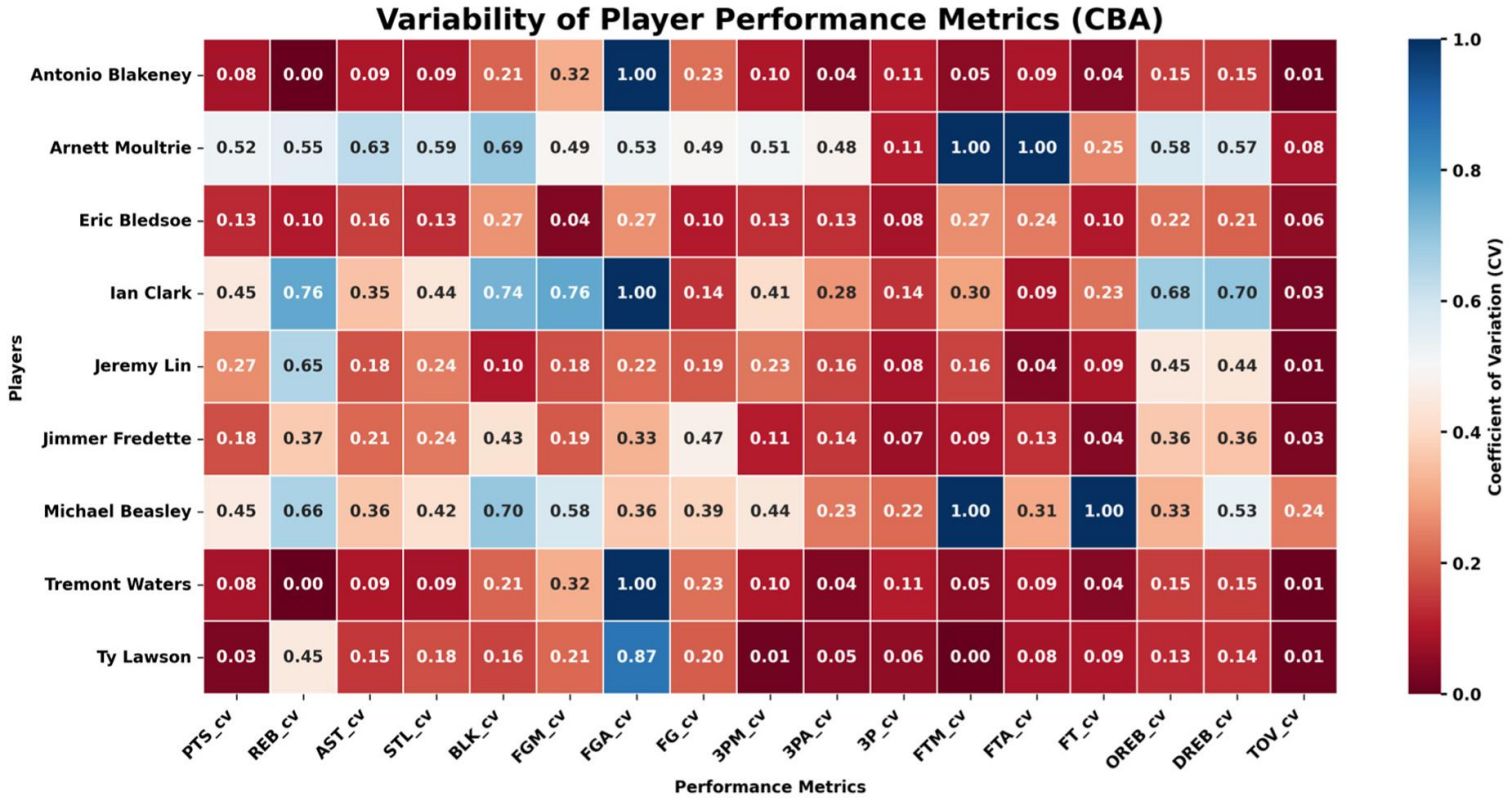
Variability of player performance metrics (CV) in CBA.

To reveal how the match between player style and league tactical structure affects performance stability when athletes cross leagues, this subsection examines NBA-to-CBA transfers such as Stephon Marbury, Jimmer Fredette, and Jeremy Lin. We compare the season-by-season coefficients of variation (CVs) for their main technical indicators before and after the move (see Figures 11, 12). The metrics cover scoring, shooting efficiency, rebounds, assists, and turnovers.

The analysis shows that, during their NBA years, these players posted uniformly low CVs across all performance dimensions, indicating high stability. After transferring to the CBA, however, perimeter and ball-dominant players exhibited markedly higher CVs in collaboration- and decision-dependent indicators such as three-point makes (3PM), three-point percentage (3P %), assists (AST), and turnovers (TOV). For instance, Fredette and Lin experienced much wider year-to-year swings in these metrics once in the CBA, suggesting that when personal style is not well aligned with a league's prevailing tactics, performance continuity is harder to maintain.

Adaptation differs by player type. Athletes whose games center on foundational skills such as rebounding and shot-blocking show smaller CV shifts. Ty Lawson, for example, registered limited variance in offensive/defensive rebounds (OREB, DREB) and blocks (BLK) during his CBA tenure, displaying strong transfer stability. This indicates that interior or fundamentally sound players can more easily sustain consistent, predictable output across different league structures, whereas perimeter players who rely heavily on team coordination are more sensitive to tactical changes and thus show larger annual oscillations.

In sum, the empirical results confirm the core logic of *player style → tactical-structure fit → performance stability*. When athletes move from an elite league to a lower-tier one, multi-dimensional performance volatility rises noticeably—especially in offense- and collaboration-related metrics such as three-point shooting, assists, and turnovers. Players with pronounced physical-confrontation skills, however, generally demonstrate stronger adaptability and steadier performance. These findings supply data-driven guidance and theoretical reference for career planning, recruiting, and talent management.

## 4 Conclusion

This study explores how player movement between the NBA and CBA affects performance stability, focusing on the fit between league-specific tactical structures and individual player styles. The results show that pronounced tactical differences between the two leagues are the primary source of cross-league adaptation challenges and performance volatility. The NBA prioritizes floor spacing, perimeter shooting, and team coordination, with highly stable role specialization—consistent with prior research (27–29). In contrast, the CBA emphasizes physical confrontation, interior finishing, and ball control, and its tactical roles and technical metrics are comparatively less stable, aligning with the findings of Li et al. (30) and Chen et al. (31).

These structural differences directly affect player-performance stability. Perimeter and ball-dominant players (e.g., Jimmer Fredette, Jeremy Lin) exhibit greater variability in assist, three-point, and turnover metrics after moving to the CBA, indicating adaptation barriers caused by style mismatch. Conversely, players who excel in interior play and rebounding (e.g., Ty Lawson) maintain more consistent cross-league performance, showing that foundational physicality and inside skills transfer more readily across systems.

Methodologically, this work integrates a Branched Multilayer Perceptron (Branch-MLP) with SHAP interpretability to uncover data-driven links between player style, tactical structure, and performance. Empirical results confirm that Branch-MLP outperforms traditional models in the NBA's well-defined tactical environment, echoing Sukumaran et al. (19) on the advantages of deep learning. Clustering analysis further clarifies the structural relationship between style and adaptability.

Based on these findings, tailored training and recruiting strategies are advisable:

1. Versatile wings and 3-and-D specialists should strengthen collaboration, floor spacing, and defensive rotations to enhance spatial adaptability.
2. Ball-dominant scorers and offensive guards should focus on decision-making, ball handling, and shooting consistency; CBA players need better perimeter offense and resilience under pressure, while NBA players should emphasize physical confrontation.
3. Role players and defensive bigs should consolidate defensive fundamentals and rebounding to improve system fit.

A training framework that targets style–structure alignment can markedly improve cross-league adaptation efficiency and performance stability.

## 5 Conclusion

This study, grounded in deep learning and interpretable analysis, systematically reveals the core differences between the NBA and CBA in tactical structure, player styles, and adaptation mechanisms. The findings show that pronounced tactical divergences across leagues directly affect the post-transfer performance stability of players: perimeter and ball-dominant players are more prone to fluctuation, whereas interior players display greater adaptability. The Branched MLP model outperforms traditional methods in tactical recognition and outcome prediction, and clustering analysis further clarifies the impact of style–fit on performance. Practically, training and recruitment strategies should be differentiated according to the alignment between player style and the target league's structure. These insights enrich the theory of performance analysis and provide empirical support for optimizing professional development and youth training systems.

Nevertheless, the study has limitations. First, it does not systematically control for confounding variables such as cross-cultural adaptation, language environment, and psychological state, all of which may influence actual performance. Second, the limited number of cross-league transfers constrains the generalizability and robustness of the conclusions. Third, performance evaluation relies mainly on technical statistics, making it difficult to capture multidimensional abilities such as biomechanics, tactical execution, and coaching decisions. Future research should expand the sample size, integrate biomechanical parameters, tactical behaviors, and coaching data, and refine performance evaluation from multiple perspectives to advance the study of cross-league adaptation mechanisms.

## Data Availability

The raw data supporting the conclusions of this article will be made available by the authors, without undue reservation.
